# Transformation of resident notochord‐descendent nucleus pulposus cells in mouse injury‐induced fibrotic intervertebral discs

**DOI:** 10.1111/acel.13254

**Published:** 2020-10-21

**Authors:** Tiffany Y. K. Au, To‐Kam Lam, Yan Peng, Sarah L. Wynn, Kenneth M. C. Cheung, Kathryn S. E. Cheah, Victor Y. L. Leung

**Affiliations:** ^1^ School of Biomedical Sciences The University of Hong Kong Hong Kong China; ^2^ Centre for Reproduction Development, and Growth Li Ka Shing Faculty of Medicine The University of Hong Kong Hong Kong China; ^3^ Department of Orthopaedics and Traumatology The University of Hong Kong Hong Kong China

**Keywords:** disc degeneration, fibroblast, fibrosis, notochord, nucleus pulposus

## Abstract

Intervertebral disc degeneration (IDD), a major cause of low back pain, occurs with ageing. The core of the intervertebral disc, the nucleus pulposus (NP), embedded in a proteoglycan‐rich and gelatinous matrix, is derived from the embryonic notochord. With IDD, the NP becomes fibrous, containing fewer cells, which are fibroblastic and of unknown origin. Here, we used a lineage tracing strategy to investigate the origin of cells in the NP in injury‐induced mouse IDD. We established a *Foxa2* notochord‐specific enhancer‐driven Cre transgenic mouse model (Foxa2mNE‐Cre) that acts only in the embryonic to foetal period up to E14.5, to genetically label notochord cells with enhanced green fluorescent protein (EGFP). When this mouse is crossed to one carrying a Cre recombinase reporter, Z/EG, EGFP‐labelled NP cells are present even at 2 years of age, consistent with their notochordal origin. We induced tail IDD in Foxa2mNE‐Cre; Z/EG mice by annulus puncture and observed the degenerative changes for 12 weeks. Soon after puncture, EGFP‐labelled NP cells showed strong *Col2a1*+ expression unlike uninjured control NP. Later, accompanying fibrotic changes, EGFP‐positive NP cells expressed fibroblastic and myofibroblastic markers such as *Col1a1*, ASMA, FAPA and FSP‐1. The number of EGFP+ cells co‐expressing the fibroblastic markers increased with time after puncture. Our findings suggest resident NP cells initially upregulate *Col2a1*+ and later transform into fibroblast‐like cells during injury‐mediated disc degeneration and remodelling. This important discovery concerning the cellular origin of fibrotic pathology in injury‐induced IDD has implications for management in disease and ageing.

## Graphical abstract

Resident notochord descendant cells may transform into collagen II expressing cells and fibroblast‐like cells in induced mouse intervertebral disc degeneration featuring disc fibrosis. This work implicates a trans‐differentiation potential of nucleus pulposus cells in response to injurious or inflammatory stress, providing insights into the cellular origin of disc related pathology.

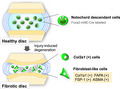

## 1 INTRODUCTION

Intervertebral disc (IVD) degeneration (IDD), a major cause of low back pain, occurs with ageing, but the pathobiology of its initiation and progression remains elusive. Studies of structural, biomechanical and matrix properties in human IDD as well as spontaneous or injury‐induced mouse models of IDD implicate fibrotic remodelling (Yang et al., 2009b; Yee et al., 2016; Zhang et al., 2018), whereas stem cell‐mediated disc repair is associated with reduced fibrotic events (Leung et al., 2014). Fibroblast activity is transiently induced after tissue injury as a normal reparative response. Chronic injury and prolonged repair may lead to unrestrained fibroblast activity and hence fibrosis without effective tissue remodelling. Fibroblasts and myofibroblasts, the activated form of fibroblasts, secrete fibrotic cytokines and chemokines that self‐stimulate and activate and recruit immune cells (Parsonage et al., 2005; Rodemann & Muller, 1991; Tomasek et al., 2002). Identifying the source of the fibroblasts and their activation is key to modifying fibrotic diseases. One major source of myofibroblasts is resident fibroblasts or mesenchymal cells (Duffield et al., 2013; Hu & Phan, 2013; Mack & Yanagita, 2015; Wynn, 2008). We tested if resident nucleus pulposus (NP) cells have a capacity to transform into fibrotic cells in IDD.

**Figure 1:**
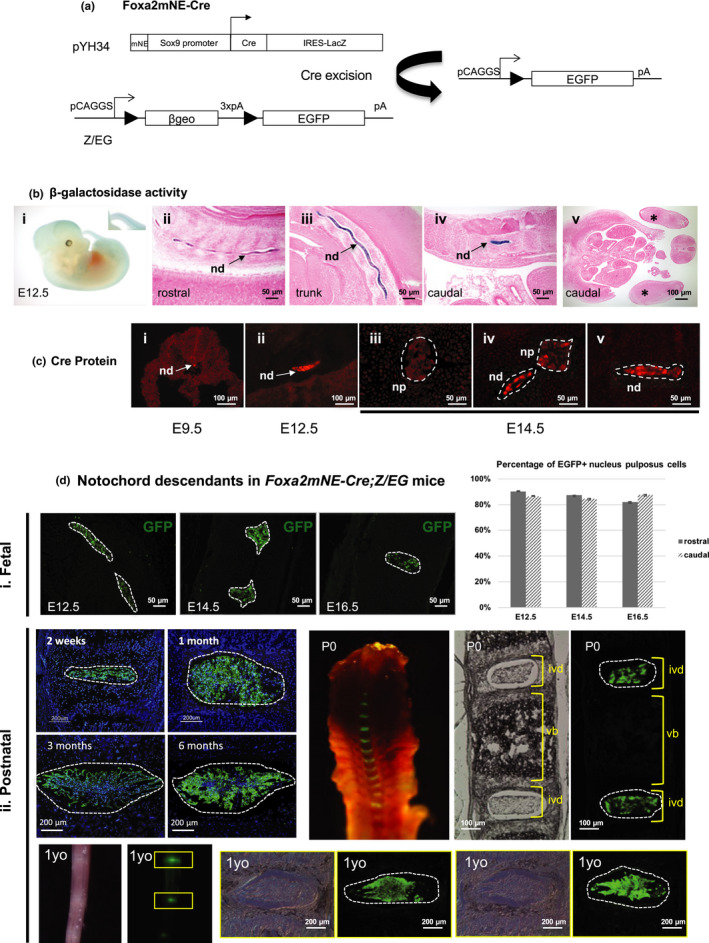
Lineage tracing in *Foxa2mNE*‐*Cre*;*Z*/*EG* mice. (a) *Foxa2* minimal notochord element (*Foxa2mNE*) and a *Sox9* minimal promoter‐driven *Cre* recombinas*e* (*Cre*) and *lacZ* via an IRES sequence. (b) β‐gal staining of *Foxa2mNE*‐*Cre* transgenic foetus at E12.5 in whole‐mount (i); mid‐sagittal sections showing notochord specificity and absence of staining in surrounding tissue (ii‐iv); mid‐cross section showing developing limbs (v,*). (c) Cre immunofluorescence in the developing notochord at E9.5 (i) and E12.5 (ii), and in (iii) rostral, (iv) trunk developing nucleus pulposus and (v) caudal notochord at E14.5 (outlined). (d) *Cre* expression and activity (EGFP) in *Foxa2mNE*‐*Cre*;*Z*/*EG* compound heterozygotes at (i) foetal stages and quantification of EGFP+ cells in the developing rostral (anterior to hindlimb) and caudal (posterior to hindlimb) nucleus pulposus (mean ± SEM < 1%); (ii) Tracking at neonatal (P0), adolescent (2 week, 1 month) and adult stage (3 months, 6 months, 1 year) by native EGFP signal detection in P0 and 1 year old tails, or EGFP immunostaining. nd, notochord; np, nucleus pulposus; vb: vertebrae; ivd: intervertebral disc; white dashed line encircled developing nucleus pulposus region

In the mouse, the NP of the IVD is formed in the disc anlagen from E12.5 onwards, as the notochord undergoes segmentation. Cre‐based tracing studies in mice show that NP cells are descendants of the notochord and may acquire a chondrocyte‐like phenotype upon ageing (Choi et al., 2008; McCann et al., 2012; Mohanty et al., 2019). *Foxa2* expression in the node/notochord is regulated by a 520 bp minimal enhancer element (mNE) (Sasaki & Hogan, 1996). We generated five independent transgenic founder lines (*Foxa2mNE*‐*Cre*) carrying a construct (Figure 1a) that expresses *Cre* under the control of the mNE and linked to an IRES‐lacZ reporter. Lines from three of these founders showed notochord expression by ß‐gal staining at E8.0 (Guo et al., in press). Therefore, we selected these three mouse lines for further characterization. Β‐galactosidase (β‐gal) staining demonstrated that the *Foxa2mNE*‐*Cre* transgene was specifically expressed, albeit in a mosaic pattern, in the developing notochord at E12.5 (Figure 1b), from rostral to caudal, recapitulating the expression pattern in the mNE‐lacZ transgenic mice (Sasaki & Hogan, 1996). Cre protein was strongly expressed in the whole developing notochord at both E9.5 and E12.5 (Figure 1c), mirroring the β‐gal activity, with expression decreasing caudorostrally at E12.5 (Figure 1b), and reducing further in the newly formed NP at E14.5 (Figure 1c, iii–v). At E14.5, LacZ expression was restricted to the notochord and was absent in the developing vertebral bodies (Figure S1a) No transgene activity was detected in skeletal elements of developing limbs at E12.5 (Figure 1b, v) and E15.5 (Figure S1b). We tested the notochord specificity of the Cre activity of the three lines by crossing *Foxa2mNE*‐*Cre* mice with *Z*/*EG* reporter mice (Novak et al., 2000) (Figure 1d). At E9.5, Cre activity reflected by enhanced green fluorescent protein (EGFP) expression was seen either only in the notochord or in the notochord and tail bud in these lines. This difference could be due to the influence of integration site and genetic background. Thus, in this study, we focused on analysing mice showing notochord/NP‐specific activity. Over 80% (mean ± SEM < 1%) of cells in the notochord expressed EGFP signal from E12.5 to post‐segmentation at E14.5 and E16.5, and rostral and caudal notochord/NP showed similar percentage of positivity (Figure 1d, i and bar chart). Strong EGFP signal was detected in the NP from 2 weeks which was present even at 15 months to 2 years of age (Figure 1d, ii and Figure S1e). No Cre expression was detected in the NP at postnatal day 9 (Figure S1c).

In human IDD, notochordal‐like cells disappear from the NP and are replaced by chondrocyte‐like and fibroblastic cells (Hunter et al., 2004; Pazzaglia et al., 1989; Trout et al., 1982). A recent tracing study using *Krt19*‐*CreERT* mice indicated NP cells could acquire a chondrocyte‐like phenotype in ageing (Mohanty et al., 2019). We used a 3‐month‐old adult mouse tail disc puncture injury model in which a cartilaginous and later fibrocartilaginous NP phenotype develops, analogous to the histological features found in moderate and advanced human IDD stages (Yang et al., 2009a). In *Foxa2mNE*‐*Cre*;*Z*/*EG* mice, unlike the adjacent uninjured discs, we observed progressive morphological changes of the IVD after injury, including the appearance of cell clusters, loss of the distinct NP:annulus boundary and NP matrix remodelling (Figure S2). Disc height and histological quality were reduced but were partially reversed in later phase (Figure S3a,b). This raises the possibility that the matrix remodelling might play roles in functional compensation, or otherwise attempt to elicit a reparative process involving a granulation tissue state, which eventually contributes to a fibrocartilaginous NP due to a lack of proper tissue regeneration.

**Figure 2:**
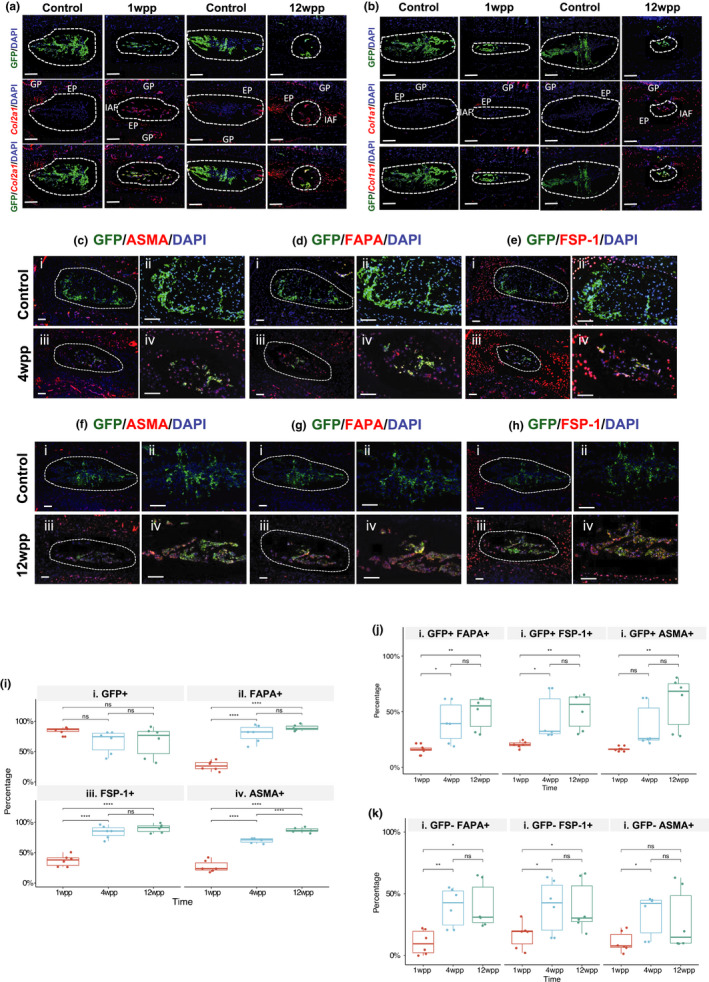
Resident NP cell transformation into fibroblast‐like cells in induced disc degeneration. (a) Combined EGFP immunofluorescence and *Col2a1* in‐situ hybridization at 1 wpp and 12 wpp. (b) Combined EGFP immunofluorescence and *Col1a1* in‐situ hybridization at 1 wpp and 12 wpp. NP region encircled. GP: growth plate; IAF: inner annulus fibrosus; EP: endplate; scale bar: 200 µm. Co‐immunolocalization of EGFP with ASMA (c), FAPA (d) and FSP‐1 (e) at 4 wpp and 12 wpp (f–h). (i) Quantification of NP cells positive for EGFP or the fibroblastic markers (>3 mice, 6 sections each timepoint). (j) Percentage of EGFP+ cells co‐expressing the fibroblastic markers. (k) Percentage of EGFP—cells co‐expressing the fibroblastic markers. *p < 0.05; **p < 0.01; ****p < 0.0001; ns: *p* > 0.05. wpp: weeks post‐puncture. Scale bar in (i & iii): 25 μm; 50 μm (ii & iv). For single staining images, see Figures S5 and S6

In the uninjured discs, EGFP expression was strongly specific to the NP. In the injured discs, up to 12 weeks post‐puncture (wpp), EGFP+ cells could be detected in the NP (Figure S3c). While untreated discs displayed a low rate of proliferation and apoptosis (Figure S3d), an increase of apoptotic activity was observed in the injured NP by 2 wpp (Figure S3e). No sign of Cre protein expression was found in the disc after puncture (Figure S4a,b). These data suggest the EGFP+ cells are descendants of notochordal/NP cells labelled in the foetal period rather than cells labelled by Cre reactivation in response to injury. Combined immunofluorescence and in‐situ hybridization indicated the appearance of EGFP+ cells strongly expressing *Col2a1* transcripts in the NP of injured discs at 1 and 12 wpp but not in the control adjacent disc (Figure 2a). Later, at 12 wpp *Col1a1* expressing EGFP+cells appeared in the NP of injured discs which were absent in the adjacent uninjured controls (Figure 2b). This finding is consistent with the hypothesis that some local NP cells survive and acquire a chondrocyte‐like and later a fibroblast‐like phenotype in injury‐induced IDD.

We investigated whether the *Col1a1*‐expressing cells have characteristics of fibroblasts and myofibroblasts, the effector cells in the proliferative stage of wound healing as well as in tissue fibrosis. Fibroblast‐specific protein 1 (FSP‐1) is a marker of quiescent fibroblasts (Quail & Joyce, 2013; Strutz et al., 1995). Activated fibroblasts express fibroblast activation protein (FAPA) (Strutz et al., 1995), while α‐smooth muscle actin (ASMA) is a marker of myofibroblasts (Kahounova et al., 2018). We found increased expression of FSP‐1, FAPA and ASMA in the NP by 4 wpp, co‐expressed by a subpopulation of the EGFP+ cells (Figure 2c–e). By 12 wpp, the expression of FSP‐1, FAPA and αSMA became more homogeneous in the NP, with many cells co‐expressing EGFP signal and fibroblastic markers (Figure 2f–h). Quantification confirmed a progressive increase of EGFP+ cells co‐expressing fibroblastic markers with time (Figure 2i,j). These findings suggest that resident NP cells can acquire a fibroblast‐ or myofibroblast‐like identity in the course of injury‐induced IDD. Notably, we also observed an increasing trend of fibroblast‐like cells that are EGFP‐negative during early degeneration (from 1 to 4 wpp) (Figure 2k). However, the EGFP+ cells (50% FAPA+; 51% FSP‐1+; 59% ASMA+) showed a higher rate of fibroblastic marker expression than the EGFP‐negative cells (40% FAPA+; 39% FSP‐1+; 28% ASMA+) at late stage (12 wpp). It is not clear if the EGFP‐negative fibroblast‐like cells are related to mosaic Cre expression (McLellan et al., 2017) or implicate non‐local sources of the fibroblastic cells. These possibilities can be investigated in the future by pulse‐chase type tracing studies using a NP‐specific inducible‐Cre system.

Our findings show that in an injury‐induced model of IDD, cells in the NP can transform into *Col2a1* expressing cells and later fibroblast‐like cells. Increasing sample size and examination of additional markers in future investigation may consolidate and capture the transformation process at better resolution. To our knowledge, this is the first in vivo report that notochord derived cells can transform to become fibroblast‐/myofibroblast‐like cells in IDD induced by injury. This important discovery concerning the cellular origin of fibrotic pathology in IDD challenges the common belief that IDD mainly involves cell infiltration from the endplate or annulus that remodels the NP and has implications for management in disease and ageing. The implication of our results is that, in response to injury, some NP cells can survive and perhaps transdifferentiate as a survival mechanism and/or as a consequence of inflammation and stress signals. Our recent discovery that the integrated stress response directly reprograms chondrocytes to a more juvenile state (Wang et al., 2018) is consistent with such a possibility. The consequence of the altered differentiation state is a change in expression of ECM genes leading to the fibrotic state featured at late‐stage degeneration. Notably, the NP cell composition of young adult mice in our study resembles to that of foetal/perinatal, instead of mature, human discs. Future cell tracing investigations in different degeneration models may provide additional insights onto the cellular origins of the remodelling process.

## CONFLICT OF INTEREST

None declared.

## AUTHOR CONTRIBUTIONS

T.Y.A., K.S.E.C. and V.Y.L. conceived and designed the study. T.Y.A. and V.Y.L. wrote the manuscript. V.Y.L and K.S.E.C. supervised the study and analysed the data. T.Y.A., T.K.L, Y.P. and S.W. performed experimental works and analysed the data. K.S.E.C. and K.M.C. edited the manuscript.

## Data Availability

Data available in article supplementary material and on request form the authors.
