# Supplementary material for: Transformation of resident notochord‐descendent nucleus pulposus cells in mouse injury‐induced fibrotic intervertebral discs
Source: Aging Cell. 2020 Oct 21;19(11):e13254. doi: 10.1111/acel.13254 (PMC7681061; doi:10.1111/acel.13254)
Supplement: Supplementary file 1 [file ACEL-19-e13254-s001.pdf]

Supporting information

Supplementary figures

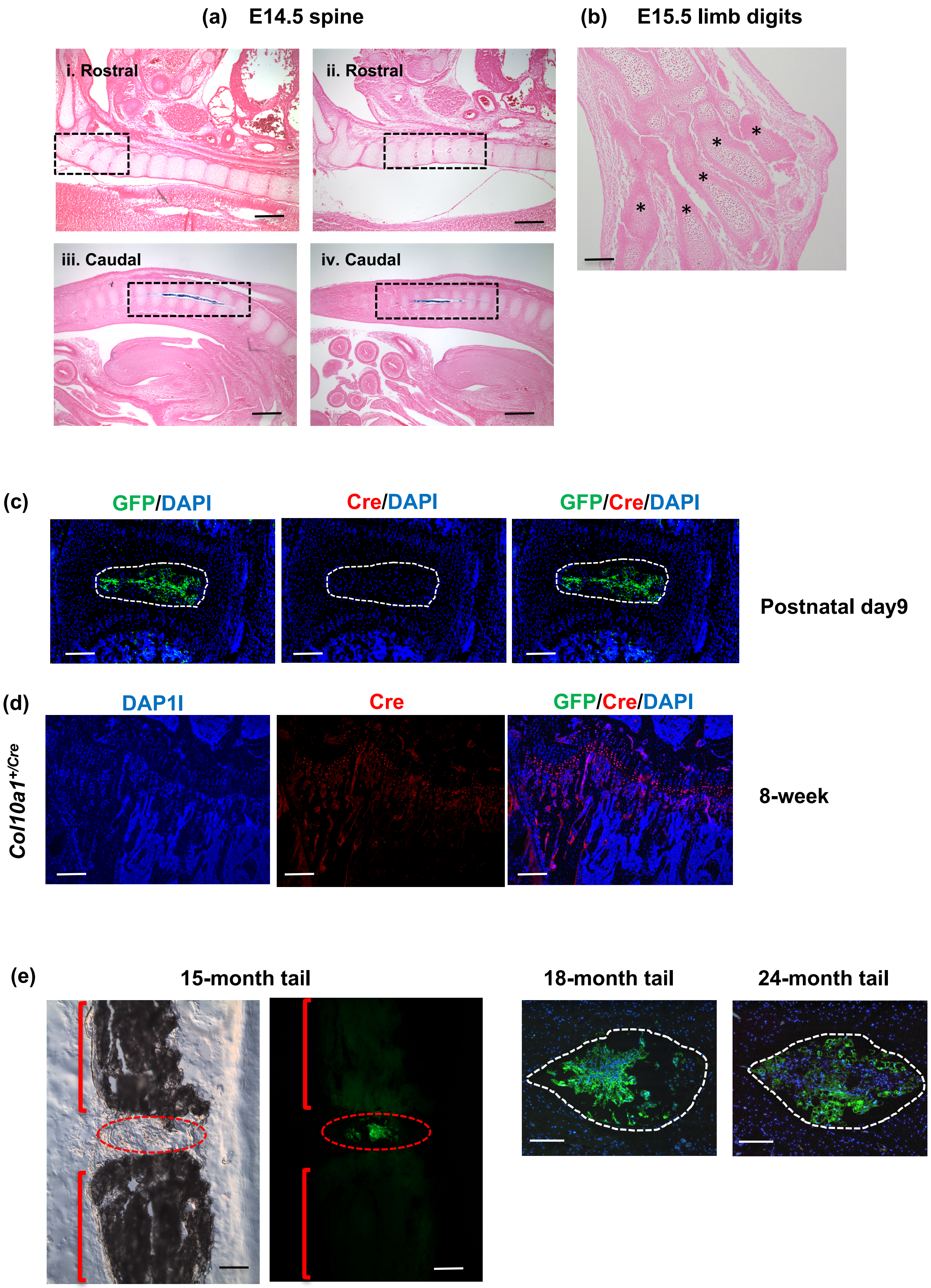

Figure S1

Foxa2mNE-Cre transgene expression was detected in fetal notochord but no Cre recombinase expression was detected at fetal developing limb or at postnatal stage day 9.

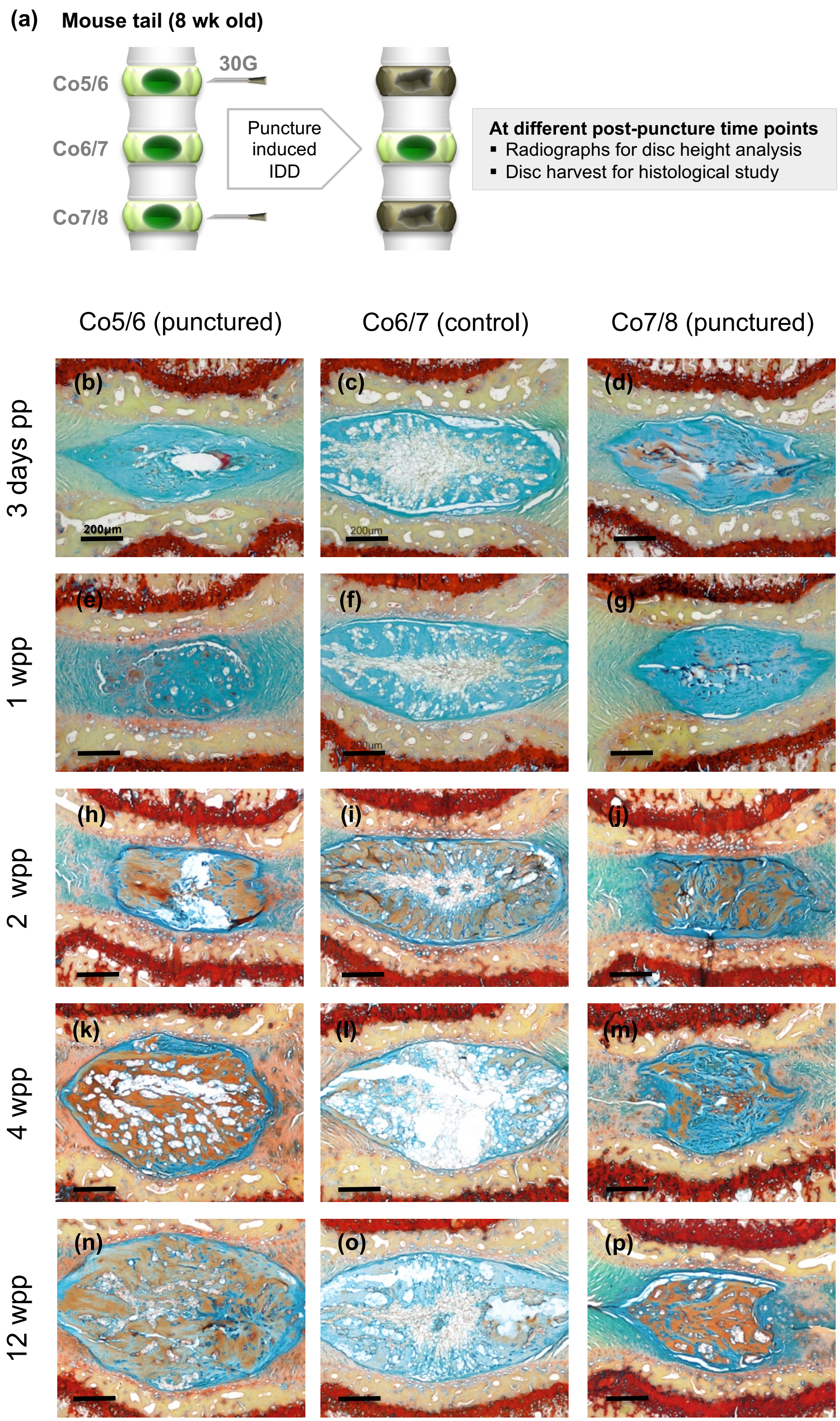

Figure S2
Morphological changes of punctured murine discs.

Variable	Feature description	Score
**NP cellularity**	Single NP cell mass	0
	Honey comb structure	1
	Decreased cellularity with cell cluster formation	2
	Significant cell loss (> ~50%)	3
	No NP cells	4
**NP matrix staining**	Predominantly Alcian blue staining	0
	Mild Safranin O staining (< ~20% area)	1
	Significant Safranin O staining (> ~20% area)	2
**AF matrix staining**	No Alcian blue staining	0
	Mild Alcian blue staining	1
	Significant Alcian blue staining (> ~50% area)	2
**Cleft/fissure**	No clefts/fissures	0
	Mild clefts/fissures	1
	Severe clefts/fissures	2
**NP-AF interface**	Intact NP-AF boundary	0
	Serpentine AF and/or appearance of large rounded cell clusters near the boundary	1
	Slightly reversed AF laminae	2
	Severely reversed AF laminae or undefined NP-AF boundary	3

Supplementary Table 1. Histological grading scheme.
Total score: 0-1 = Grade 0 ; 2-4 = Grade 1 ; 5-7 = Grade 2 ; 8-10 = Grade 3 ; >11 = Grade 4. NP: nucleus pulposus; AF: annulus fibrosus

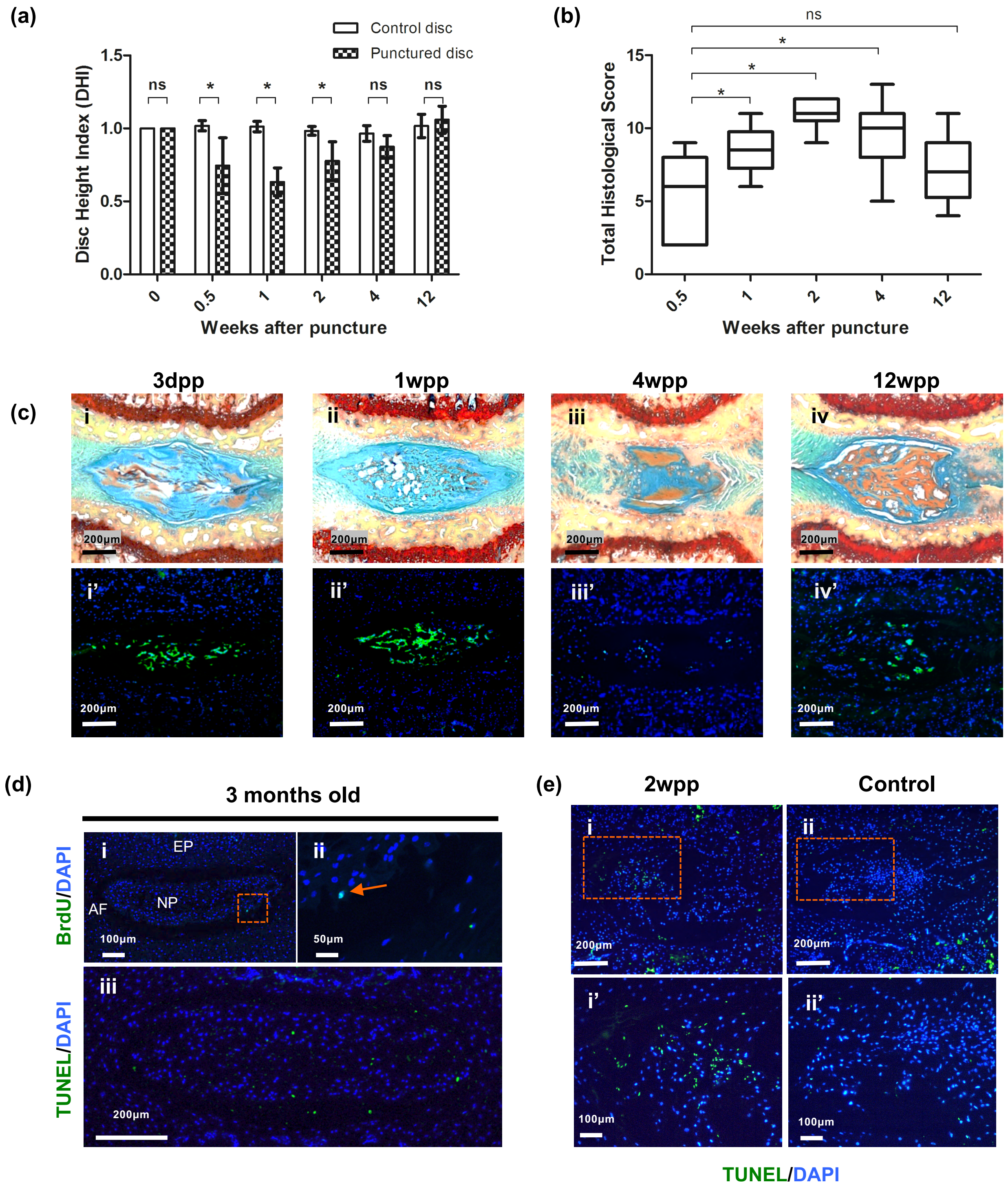

Figure S3

Progression of induced disc degeneration and NP cell fate.

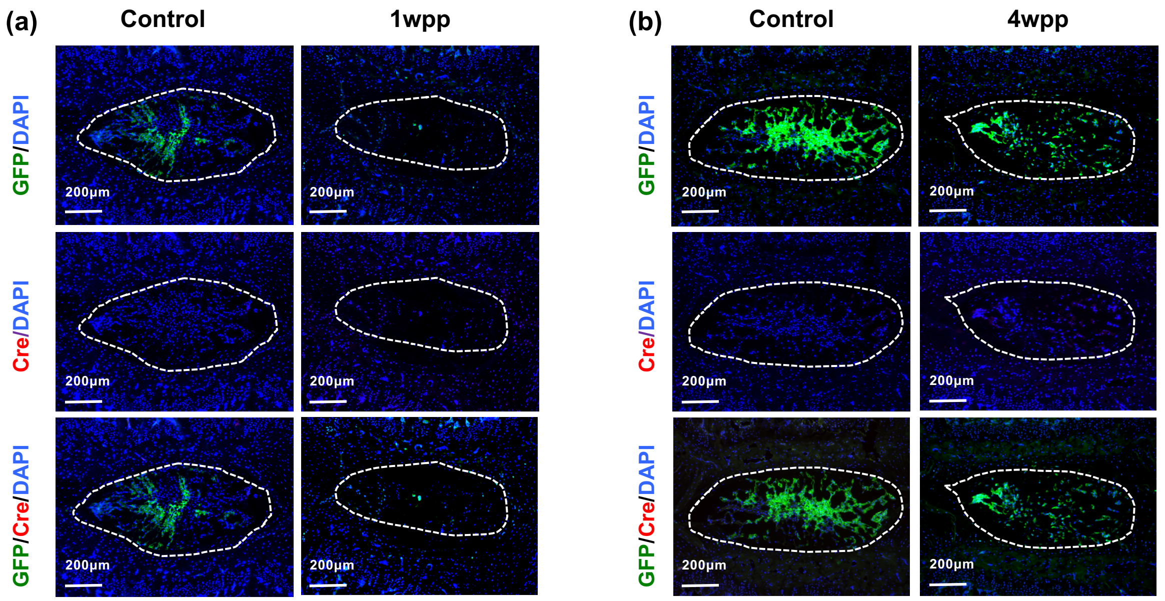

Figure S4

No Cre recombinase expression was detected or induced at postnatal stages with/without puncturing, including 1wpp (a) and 4wpp (b).

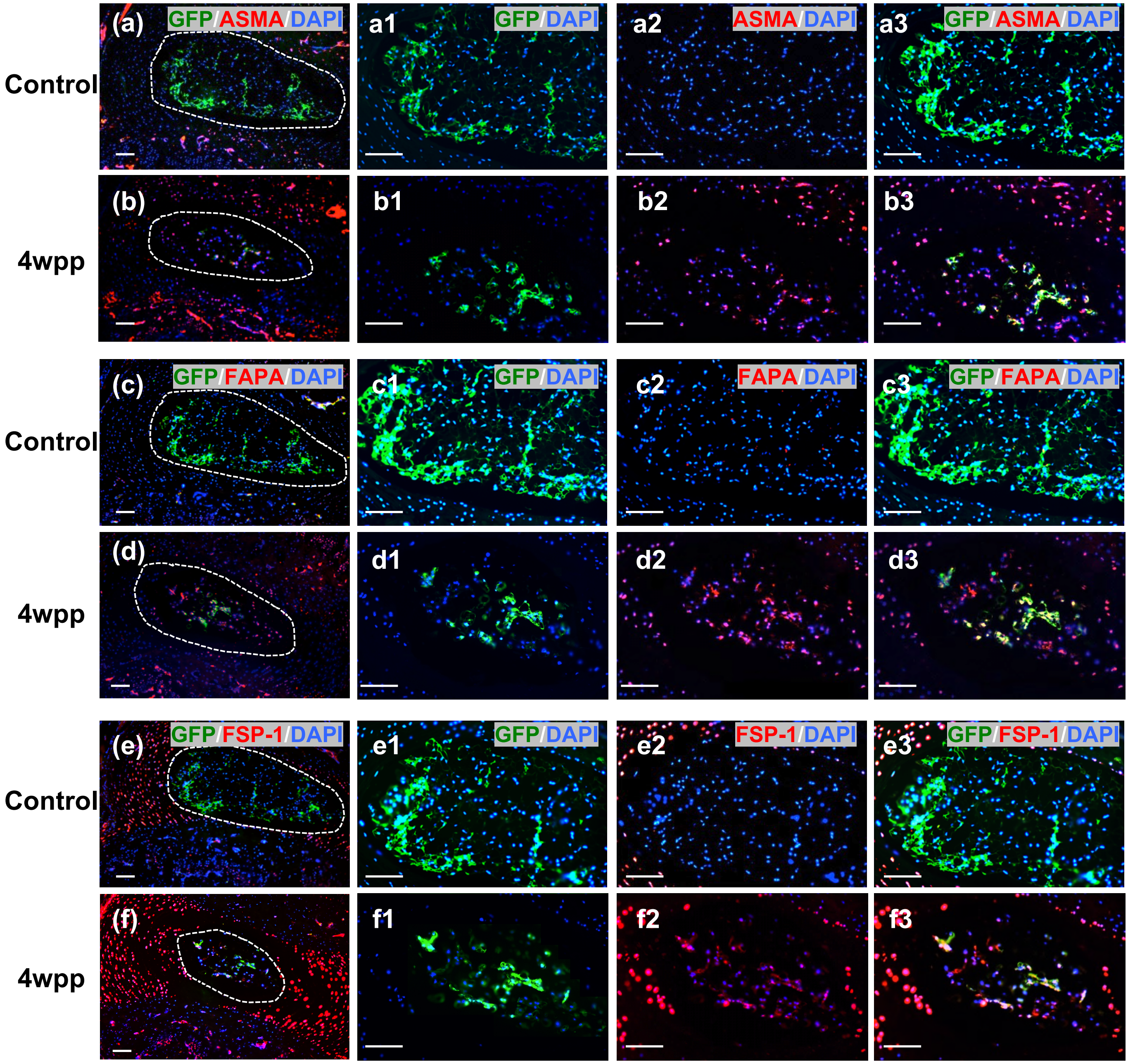

Figure S5

Resident NP cell differentiation into fibroblast-/myofibroblast-like cells in induced disc degeneration at 4 weeks post-puncturing.

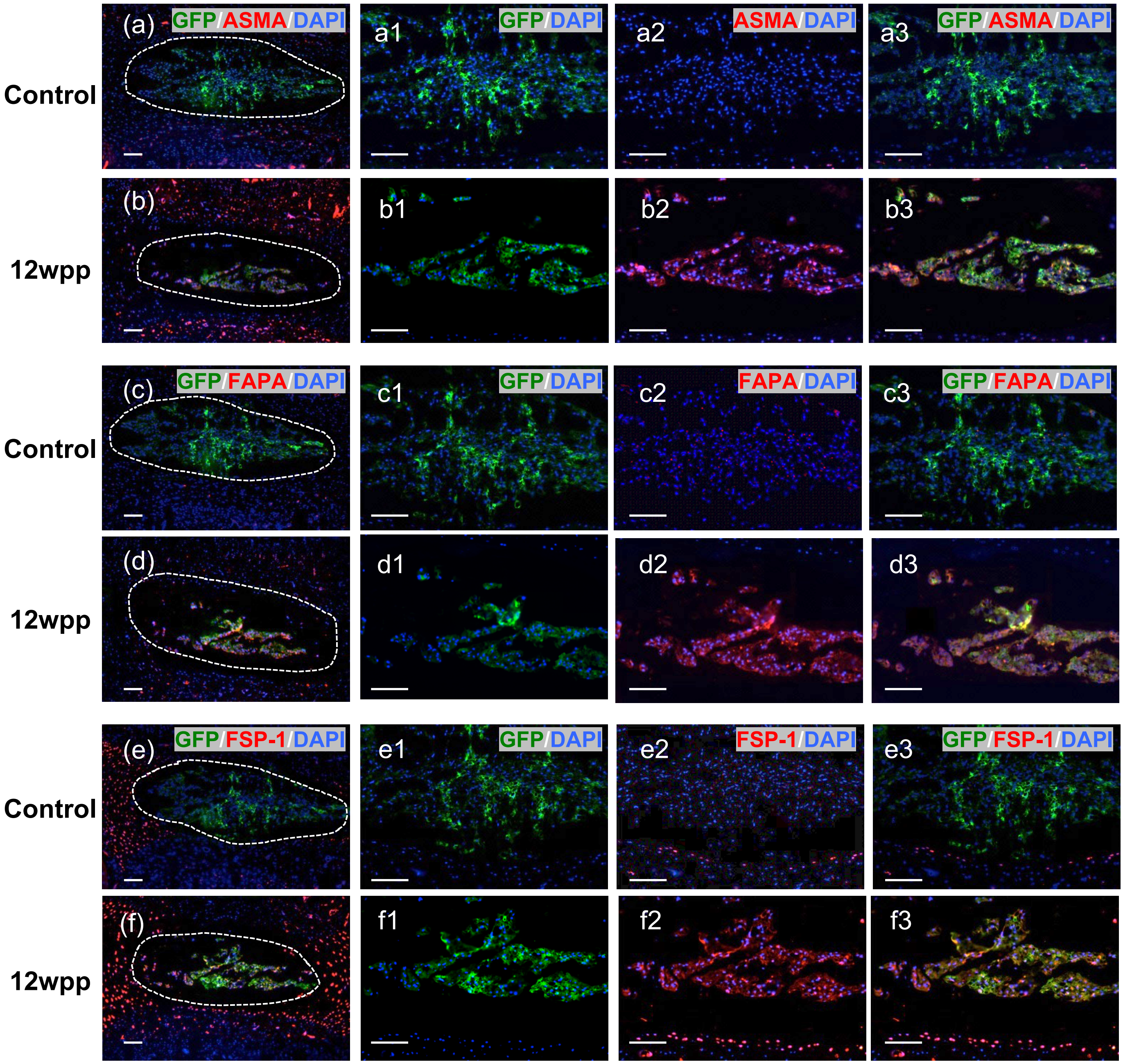

Figure S6

Fibroblast-/myofibroblast-like cells accumulation in induced disc degeneration at 12 weeks post-puncturing
