# Supplementary material for: Transformation of resident notochord‐descendent nucleus pulposus cells in mouse injury‐induced fibrotic intervertebral discs
Source: Aging Cell. 2020 Oct 21;19(11):e13254. doi: 10.1111/acel.13254 (PMC7681061; doi:10.1111/acel.13254)
Supplement: Supplementary file 2 [file ACEL-19-e13254-s002.docx]

**“Transformation of resident notochord-descendent nucleus pulposus cells in mouse injury-induced fibrotic intervertebral discs” by Au T, et al.**

**Supporting information**

**Experimental Procedures**

**Generation of *Foxa2mNE-Cre* transgenic mice**

To generate the Foxa2mNE-Cre transgene (pYH34), the 520bp minimal notochord enhancer of the mouse Foxa2 gene (a gift of H.Sasaki) was cloned upstream of the genomic *Sox9* (gift of P.Koopman), followed by replacing the coding region of *Sox9* with the *Cre* recombinase gene (isolated form pBS185, GibcoBRL). An IRES-*lacZ* reporter (gift of D.Wilkinson.) was inserted between the *Cre* Stop codon and the *Sox9* polyadenylation sequence. The 10.8kb insert was released from the vector backbone by *Not1* and *Sal1* digestion, purified, and then microinjected into the pronuclei of fertilized C57BL/6 x CBA F1 hybrid eggs to generate transgenic mice. Founder mice were identified by polymerase chain reaction (PCR) performed on ear biopsies using Cre-specific primers 5’-GGACATGTTCAGGGATCGCCAGGCG-3’ and 5’-GCATAACCAGTGAAACAGCATTGCTG-3’. The *Foxa2mNE-Cre* transgenic mouse lines were maintained on the C57BL/6 background. The male transgenic *Foxa2mNE-Cre* mice were kept for breeding with female C57BL/6 mice.

**Validation of transgene activity and lineage tracing**

Whole-mount β-gal staining of mouse embryos at various pre-natal stages was performed as described earlier (Hogan et al., 1994) to examine the lacZ reporter expression in the *Foxa2mNE-Cre* transgenic mice. Briefly, E7.5-E12.5 embryos were fixed in 4% paraformaldehyde (PFA) at 4˚C for 3-15 minutes, then washed in rinse buffer (10% NP40 and 1% deoxycholate in PBS) three times for 10 minutes each. The samples were finally incubated in the washing buffer supplemented with 1mg/ml X-gal, 2mM MgCl_2_, 5mM EGTA, 5mM potassium ferrocyanide, and 5mM potassium ferricyanide for 2 hours to overnight at 37˚C.

To study the Cre activity, the *Foxa2mNE-Cre* founders were crossed with Z/EG homozygous reporter mice (Novak et al., 2000). The yolk sac of prenatal mice/ skin biopsies of postnatal mice was digested by proteinase K and the genomic DNA was extracted for genotyping. Enhanced green fluorescent protein (EGFP) expression in the *Foxa2mNE-Cre;Z/EG* mice was examined by live fluorescent imaging and immunostaining. To assess the Foxa2mNE-Cre descendent cells in fetal discs, EGFP expression was evaluated by immunostaining at E12.5, E14.5 and E16.5 (n=3). For each animal, NPs from at least 6 different disc levels were examined and number of EGFP positive cells and DAPI positive cells were counted.

**Induction of mouse tail disc degeneration**

To investigate the fate of notochord descendent cells in disc degeneration, annulus puncture was performed in the tail discs of *Foxa2mNE-Cre;Z/EG* double transgenic mice as previously described (F. Yang, Leung, Luk, Chan, and Cheung (2009)) under the approval of local animal ethics committee. Briefly, 3-month-old mice (n=18), of mixed gender, were anesthetized by intraperitoneal injection of Hypnorm and Dormicum at 1ml/kg of body weight and the caudal disc levels were identified by X-ray (Model 43 855a; Faxitron Corp, IL, USA). The tail skin was incised longitudinally and the Co5/6 and Co7/8 coccygeal levels were punctured by inserting a 30G needle (BD biosciences) into the dorsal annulus perpendicular to middle of the disc, at 1mm depth controlled by a needle forceps holder, under the guidance of surgical microscope (Wild M691, Switzerland). The Co6/7 level was left untreated as control. The mice were allowed to recover and have free activity in cage. The operated animals were subjected to X-ray at specified time points after puncture for disc height measurement. A subgroup of the animals was euthanized and the spine was decalcified by EDTA and embedded in paraffin for histological analysis.

**Disc height measurement**

To assess the change of disc height after induced disc degeneration, antero-posterior X-rays were taken before and after the annulus puncture under sedation. Disc height index (DHI) was determined as previously described (Masuda et al., 2005) (Masuda et al., 2005), which was calculated by averaging the lengths of anterior, middle, and posterior portions of the disc with the lengths of the anterior and posterior vertebrae. The change in the disc height was expressed as the ratio of postoperative DHI to preoperative DHI. A total of 18 mice were examined.

**Histological analysis and grading**

FAST staining was used to examine the IVD structure and assess the degeneration status during aging and induced degeneration as in previous study (Leung et al., 2009) with minor modification. (Leung, Chan, Hung, Cheung, & Chan, 2009). Briefly, the sections were dewaxed, rehydrated, and were sequentially stained with 1% Alcian blue 8GX (pH 1.0) for 1 min; then with 0.1% Safranin-O for 3 min; differentiated in 25% ethanol for 15 sec; 0.25% Tartrazine for 15 sec; and finally 0.01% Fast Green solution for 1 min (all from Sigma-Aldrich). The sections were air-dried and mounted in DePeX (BDH Laboratory; Poole, UK), and then examined under a Nikon Eclipse 80i microscope (Tokyo, Japan).

A grading system was adopted from our previous study (F. Yang et al., 2009) with slight modifications to measure the degenerative changes in the operated discs. It was based on five criteria in regard to the cellular organization and FAST profiles of the discs: (i) NP cellularity; (ii) NP staining pattern; (iii) AF staining pattern; (iv) cleft/ fissure formation and (v) NP/ AF boundary. Disc images were randomized and scored by 3 different observers. The total histological score for each disc was presented as the sum of scores from the five criteria. In total, 9 mice (18 punctured discs and 9 untreated control discs) were examined.

**Analysis of cell proliferation and apoptosis**

BrdU (5-bromo-2'-deoxyuridine) labeling was performed to examine cell proliferation in the discs (Guo, Chung, Kondo, Bringhurst, & Kronenberg, 2002). BrdU was administered to the mice by intraperitoneal injection at 200ug/g of body weight at 3 hours before harvesting the discs and the BrdU labeled cells were identified by immunostaining. TUNEL (Terminal deoxynucleotidyl transferase dUTP nick end labeling) assay (Roche) was performed according to the manufacturer’s instructions to identify apoptotic cells.

**Immunostaining**

Mid-sagittal paraffin sections were dewaxed and rehydrated. Cre protein expression in the transgenic *Foxa2^mNE^-Cre* mice (pre-natal stages) was characterized by immunostaining using rabbit anti-Cre antibody (PRB-106C, Covance, 1:400 dilution). EGFP expression in the *Foxa2^mNE^-Cre;Z/EG* mice was examined by immunostaining using rabbit anti-EGFP antibody (ab290, Abcam, 1:400 dilution). BrdU-labelled cells were examined using mouse monoclonal anti-BrdU antibody (ab136650, Abcam, 1:100 dilution). The sections were incubated with the primary antibodies at 4^o^C overnight and subsequently Alexa Fluor® 488 Donkey anti-rabbit IgG (A21206, Invitrogen) or Rhodamine Red-X goat anti-mouse IgG for 1 hour at RT. The sections were counterstained with DAPI mounting medium (Vector laboratories, inc., US) and examined under fluorescence microscope (Nikon Eclipse 80i, Japan).

To characterize the expression of myofibroblast markers, analyses were similarly performed except that the rehydrated sections were treated with Proteinase K (15µg/ml in TE buffer) for 15min at RT for antigen retrieval, followed by incubation with protein-blocking buffer (Dako North America, Inc). The sections were then incubated with the EGFP antibody (ab13970, abcam) along with antibody against aSMA (ab5694, Abcam), FAPa (ab28244, Abcam), or FSP-1 (ab27957,Abcam) at 1:500 dilution overnight at 4 ^0^C, followed by Alexa Fluor® 488 goat anti-rabbit IgG (A11012, Invitrogen) and Alexa Fluor® 594 goat anti-chicken IgG (sc-362262, Santa Cruz and ab150176, abcam) for 1 hour at RT. Counting of EGFP+aSMA+ or EGFP+FAPA+ or EGFP+FSP-1+ dual-stained cells to total EGFP+ cells was performed in six different fields of view at 20x magnification captured from each of 3 mouse samples.

***In situ* hybridization**

*In situ* hybridization was performed on 1wpp and 12wpp paraffin sections using DIG-labelled *Col2a1/Col1a1* riboprobes, followed by immunostaining of EGFP on the same

section as previously described with modification (F. Yang et al., 2009a; F. Yang, Leung, Luk, Chan, & Cheung, 2009b). After riboprobe hybridization, sections were incubated with DIG antibody conjugated with HRF (abcam, ab6216) at 1:500 dilution at room temperature for 1 hr, followed by signal amplification using TSA^TM^ Plus system with Cyanine 3 (PerkinElmer). EGFP immunostaining was then performed as described above.

**Statistical analysis**

For quantitative analysis of immunolabelled cells, ANOVA followed by post-hoc paired t-test was applied to test the significance of difference among different timepoints for each marker. One-way ANOVA followed by paired t-test was used to examine the significance of difference in DHI. One-way ANOVA followed by Bonferroni’s Test was used to examine the significance of difference in histological grades. A P value <0.05 was considered statistically significant.

**Supplementary Figures**

**Figure S1**

*Foxa2mNE-Cre* transgene expression was detected in fetal notochord and no Cre recombinase expression was detected at fetal developing limb or at postnatal stage day 9. Β-galactosidase (β-gal) staining of *Foxa2mNE-Cre* transgenic fetus at (a) E14.5 spine and (b) E15.5 limb. (a) LacZ signal was detected in developing notochord at caudal region (iii & iv) but not at rostral nucleus pulposi (NP) (i & ii). (b) No lacZ signal was detected in digit chondrocytes. *: developing digits. (c) No Cre protein was detected at postnatal day 9 NP (white dotted circle). EGFP+ cells were the *Foxa2mNE-Cre;Z/EG* descendants. (d) The specificity of the Cre antibody, PRB-106C (Covance), was shown in 8-week *Col10a1^Cre/+^* growth plate in developing limbs (L. Yang, Tsang, Tang, Chan, & Cheah, 2014). (e) Native EGFP signal detection in 15-month *Foxa2mNE-Cre;Z/EG* mouse tail. EGFP positive cells were found in the NP (red dotted circle) but not in the vertebra (red brackets). EGFP positive cells were detected by immunostaining in 18- and 24-month tail NP (white dotted circle). Scale bar: 200µm.

**Figure S2**

Morphological changes of punctured murine discs. (a) Schematic diagram showing the design of mouse tail disc annulus puncturing experiment in *Foxa2mNE-Cre;Z/EG* mice. (b-p) Histology of punctured (Co5/6 and Co7/8) and control (Co6/7) mouse tail discs. Representative FAST staining of murine caudal IVDs at different time points after annulus puncture. pp: post-puncture; wpp: week post-puncture; Scale bar in (b-p): 200µm.

**Figure S3**

Progression of induced disc degeneration and NP cell fate. (a) Changes in disc height index (DHI) after annulus puncture in tail discs of *Foxa2mNE-Cre;Z/EG* mice. The changes in the disc height after annulus puncture are indicated by the ratio of postoperative DHI to preoperative DHI. The DHI punctured samples were shown and compared with the corresponding control. ‘*’ denotes p<0.01 and ‘ns not significant. (b) Total histological scores (based on Suppl.Table 1) of the murine caudal IVDs after puncture. The score was expressed as the average (solid line in the box), ‘*’ denotes p<0.05 in comparison to the 0.5wpp score. (c) Upper panel (i-iv): Representative FAST staining showing histological and matrix changes after puncture-induced degeneration. Lower panel (i’-iv’): EGFP-labelled cells in corresponding samples. (d) Proliferation and apoptosis in caudal disc of 3 months old mice were examined by BrdU-labeling (i & ii (higher magnification)) and TUNEL assay (iii) respectively. (e) TUNEL assay in punctured mouse disc (i) at 2 wpp compared to control level (ii) (i’ & ii’ higher magnification). The dotted line encircled the NP region. dpp: days post-puncture, wpp: weeks post-puncture.

**Figure S4**

No Cre recombinase expression was detected or induced at postnatal stages with/without puncturing, including 1wpp (a) and 4wpp (b). EGFP+ cells were *Foxa2mNE-Cre* descendants in *Foxa2mNE-Cre;Z/EG* mice. The dotted line encircled the NP region. wpp: weeks post-puncture.

**Figure S5**

Resident NP cell differentiation into fibroblast-/myofibroblast-like cells in induced disc degeneration at 4 weeks post-puncturing. (a-b) EGFP-tagged notochord descendants were retained in normal and punctured NP. Myofibroblastic marker, ASMA expression was significantly increased in the punctured discs, in which co-localization of EGFP and ASMA was detected (b1-b3). (c-f) Increased expression of fibroblastic markers, FAPA (d1-d3) and FSP-1 (f1-f3), were also observed in the punctured discs. Scale bar in a, b, c, d, e, f: 25um; in a1-a3, b1-b3, c1-c3, d1-d3: 50um. Blue: DAPI labeling the nuclei; Green: immunostaining for EGFP; Red: immunostaining for ASMA, FAPA and FSP-1. wpp: weeks post-puncture.

**Figure S6**

Fibroblast-/myofibroblast-like cells accumulation in induced disc degeneration at 12 weeks post puncturing. (a-b) EGFP-tagged notochord descendants were retained in normal and punctured NP. Significant increase in ASMA expression was found in the punctured discs. EGFP+ cells co-localized with ASMA expression in punctured discs (b1-b3). (c,d) EGFP+ cell co-localization with FAPA expression (d1-d3). (e,f) EGFP+ cell co-localization with FSP-1 expression (f1-f3). Scale bar in a, b, c, d, e, f: 25 um; in a1-a3, b1-b3,c1-c3,d1-d3: 50um. Blue: DAPI labeling the nuclei; Green: immunostaining for EGFP; Red: immunostaining for ASMA, FAPA and FSP-1. wpp: weeks post-puncture.

**Supplementary Table 1.**

Histological grading scheme. NP: nucleus pulposus; AF: annulus fibrosus

**References**

Guo, J., Chung, U. I., Kondo, H., Bringhurst, F. R., & Kronenberg, H. M. (2002). The PTH/PTHrP receptor can delay chondrocyte hypertrophy in vivo without activating phospholipase C. *Dev Cell, 3*(2), 183-194.

Leung, V. Y., Chan, W. C., Hung, S. C., Cheung, K. M., & Chan, D. (2009). Matrix remodeling during intervertebral disc growth and degeneration detected by multichromatic FAST staining. *J Histochem Cytochem, 57*(3), 249-256. doi:10.1369/jhc.2008.952184

Masuda, K., Aota, Y., Muehleman, C., Imai, Y., Okuma, M., Thonar, E. J., . . . An, H. S. (2005). A novel rabbit model of mild, reproducible disc degeneration by an anulus needle puncture: correlation between the degree of disc injury and radiological and histological appearances of disc degeneration. *Spine (Phila Pa 1976), 30*(1), 5-14.

Yang, F., Leung, V. Y., Luk, K. D., Chan, D., & Cheung, K. M. (2009). Injury-induced sequential transformation of notochordal nucleus pulposus to chondrogenic and fibrocartilaginous phenotype in the mouse. *J Pathol, 218*(1), 113-121. doi:10.1002/path.2519

Yang, L., Tsang, K. Y., Tang, H. C., Chan, D., & Cheah, K. S. (2014). Hypertrophic chondrocytes can become osteoblasts and osteocytes in endochondral bone formation. *Proc Natl Acad Sci U S A, 111*(33), 12097-12102. doi:10.1073/pnas.1302703111
